# Little cigars and cigarillos harbor diverse bacterial communities that differ between the tobacco and the wrapper

**DOI:** 10.1371/journal.pone.0211705

**Published:** 2019-02-22

**Authors:** Suhana Chattopadhyay, Eoghan M. Smyth, Prachi Kulkarni, Kelsey R. Babik, Molly Reid, Lauren E. Hittle, Pamela I. Clark, Emmanuel F. Mongodin, Amy R. Sapkota

**Affiliations:** 1 Maryland Institute for Applied Environmental Health, University of Maryland School of Public Health, College Park, Maryland, United States of America; 2 Institute for Genome Sciences and Department of Microbiology and Immunology, University of Maryland, School of Medicine, Baltimore, Maryland, United States of America; 3 Department of Behavioral and Community Health, University of Maryland, School of Public Health, College Park, Maryland, United States of America; Oklahoma State University, UNITED STATES

## Abstract

Despite their potential importance with regard to infectious and chronic diseases among tobacco users, microbial constituents of tobacco products lack characterization. Specifically, to our knowledge, there are no data describing the bacterial diversity of little cigars or cigarillos. To address this knowledge gap, we tested four brands of little cigars and cigarillos. Tobacco and wrapper subsamples (n = 132) were separately subjected to DNA extraction, followed by PCR amplification of the V3V4 hypervariable region of the 16S rRNA gene, and sequencing using Illumina HiSeq. Sequences were analyzed using QIIME and Phyloseq implemented in R. We identified 2,681 operational taxonomic units across all products. Significant differences in alpha and beta diversity were observed between Swisher Sweets and Cheyenne products. Alpha and beta diversity was also significantly different between tobacco and wrapper subsamples within the same product. Beta diversity analyses of only tobacco samples identified no significant differences in the bacterial microbiota of different lots of the same products; however, the microbiota in the wrapper differed significantly across lots for all brands. Overall, *Firmicutes* were found to dominate in the wrapper, whereas *Proteobacteria* were most abundant in the tobacco. At the genus level, *Bacillus* and *Lactobacillus* dominated in the wrappers, and *Staphylococcus* and *Pseudomonas* dominated in the tobacco. Our findings suggest that the bacterial microbiota of little cigars and cigarillos is diverse and differs significantly between the tobacco and the wrapper, and across brands. Future work is necessary to evaluate the potential public health implications of these findings.

## Introduction

Cigars are a heterogeneous category of combustible tobacco products that includes little cigars, cigarillos and traditional (or large) cigars. Little cigars are similar in size to cigarettes, however, the tobacco of little cigars is wrapped in brown paper that typically contains some tobacco leaf, and little cigars are produced with or without a filter [1]. Little cigars provide a similar feel and draw to the user as smoking a cigarette; however, cigarillos (or ‘seven minute cigars’) are more like traditional cigars, containing about 3g of tobacco wrapped in tobacco leaves or brown paper mixed with tobacco leaves [1]. Cigarillos are thinner and smaller in diameter than a traditional cigar but longer than a normal cigarette [2], and, unlike traditional cigars, cigarillos are smoked with a deeper inhalation [3].

Little cigars and cigarillos are taxed lower than cigarettes across most of the US, making them a potentially more affordable product compared to cigarettes [4]. Little cigars and cigarillos can also be lawfully sold as “singles” or “loose” without health warnings compared to “packages of 20” cigarettes [5]. In addition, Kostygina et al. (2016) recently showed how flavoring of little cigars and cigarillos appeals to a larger consumer market, attracting young adults, women, low-income minority groups and non-users [6]. In 2013, roughly 12.4 million people (12 years or older) in the U.S. smoked cigars, and between 1995 and 2008 there was a 316% and 255% rise in sales of little cigars and cigarillos, respectively [7].

Given the increasing sales and consumption of little cigars and cigarillos [8,9], cigar smoking has been linked to an increasing number of health issues [10,11], ranging from coronary heart disease and chronic pulmonary diseases to cancers of the lungs [12], pharynx, esophagus, pancreas and bladder [13]. Compared to cigarettes, higher concentrations of nitrogen oxides, ammonia and carcinogenic compounds (e.g. tobacco-specific nitrosamines (TSNAs)) have been detected in the smoke of traditional cigars and play a role in the development of adverse health outcomes among users [10]. In addition, little cigars and cigarillos contain 2–5 times the amount of nicotine compared to cigarettes [14] and are inhaled rather than smoked like a cigar, increasing users’ exposures to chemical constituents. Through these deep inhalations, users of little cigars and cigarillos could also be chronically exposed to microbial constituents that could play a role in the development of both acute and chronic illnesses.

Previous studies by our group and others have characterized a wide array of bacterial populations within the tobacco microenvironment of mentholated and non-mentholated cigarettes [15–17] and smokeless tobacco products [18,19]. In cigarette products, we have shown that different brands are characterized by significantly different bacterial communities. We have also demonstrated that mentholation is correlated with a reduction in potential human bacterial pathogens [16] and selection for bacterial members that are resistant to harsher environmental conditions. Within smokeless tobacco products, we have detected viable and highly diverse bacterial populations predominantly belonging to the phyla *Firmicutes*, *Proteobacteria* and *Actinobacteria* [15,17].

However, to our knowledge, there are no available data regarding bacterial constituents of little cigars and cigarillos and their impacts on public health. This critical knowledge gap limits our understanding of microbial-related health risks associated with little cigar and cigarillo use. Therefore, in this study we characterized the bacterial community composition of little cigars and cigarillos and focused on whether bacterial communities differed between the tobacco and wrappers of these products.

## Materials and methods

### Sample collection

Little cigars and cigarillos were purchased from online vendors in the Spring of 2015. Four different brands were included: 1) Swisher sweets cigarillo natural sweet (SSO, Swisher International Inc., Jacksonville, FL, USA); 2) Swisher sweets little cigars sweet cherry flavor (SSC, Swisher International Inc., Jacksonville, FL, USA); 3) Cheyenne full flavor 100’s (CFF, Cheyenne International LLC. Grover, NC, USA); and 4) Cheyenne menthol flavor 100’s (CMB, Cheyenne International LLC. Grover, NC, USA). Swisher and Cheyenne products were selected for testing because they make up the highest percentages of the market share of cigarillos and little cigars, based on tobacco sales data reported by the Nielsen Research Company [20]. Tobacco and wrapper from three lots of SSO, CFF and CMB, and two lots of SSC were tested in replicates of six, for a total of 132 samples (66 tobacco and 66 wrapper samples).

### DNA extraction

Total DNA was extracted from all tobacco and wrapper subsamples using the same procedures as published previously [21,22]. Briefly, each product was opened under sterile conditions, and for each little cigar and cigarillo tested, the tobacco was separated from the wrapper and processed separately. For each little cigar, 0.2g of either tobacco or wrapper was weighed out separately, and total DNA was extracted from each component separately using a protocol published previously [15,16]. DNA was further purified from the lysate using the QIAmp DSP DNA mini kit 50, v2 (Qiagen, CA) using the recommended manufacturer’s protocol.

### 16S rRNA gene PCR amplification and sequencing

PCR amplification of the 16S rRNA gene and sequencing was performed using a process described previously [15,16]. Briefly, the V3V4 region of the 16S rRNA gene was amplified using 319F (ACTCCTACGGGAGGCAGCAG) and 806R (GGACTACHVGGGTWTCTAAT) universal primers barcoded for each sample that also included a linker sequence required for Illumina HiSeq 300 bp paired-ends sequencing, and a 12-bp heterogeneity spacer index sequence aimed at minimizing biases associated with low-diversity amplicon sequencing [15,16]. 50 ng of template DNA was added with Phusion High-Fidelity DNA polymerase (Thermo Fisher, USA) in a total volume of 25μl master mix along with an additional 0.375μl of Bovine Serum Albumin (BSA) (20 mg/ml, Sigma, MO, USA) as previously described [15,16]. PCR reactions using template DNA and negative controls were run under thermocycler parameters described previously [15]. Amplicons were pooled (25ng of 16S PCR amplicon from each sample) using the SequalPrep Normalization Plate Kit (Invitrogen Inc., CA, USA), and sequenced on the Illumina HiSeq (Illumina, San Diego, CA).

### Quality control and analysis of 16S rRNA gene sequences

Following sequencing, the 16S rRNA reads were initially screened for low quality and short read lengths. Screened paired reads were then assembled using PANDAseq [23], demultiplexed, and chimera trimmed using UCHIME [24] de-novo implemented in Quantitative Insights Into Microbial Ecology (QIIME; release v. 1.9) [25]. Operational Taxonomic Units (OTUs) were then clustered de-novo using VSEARCH [26] and taxonomies assigned using the SILVA database [27] (release 132) in QIIME using a minimum confidence threshold of 0.97 for taxonomic assignments. The resulting BIOM-formatted OTU table was then imported into R using RStudio (v.0.99.473). Prior to visualization with ggplot2 package [28] (v. 2.2.1) in R, sequences assigned to chloroplasts and *Cyanobacteria* were removed from further downstream analyses, as these most likely represent sequences amplified from tobacco DNA. Alpha diversity (Observed number of species and Shannon Index) [29] was calculated on non-normalized non-rarefied and rarefied datasets, and tested for significance with the TukeyHSD test in R using phyloseq [30] (v. 1.19.1). To account for uneven sequencing depths across samples in subsequent analyses, the OTU table was normalized using MetagenomeSeq’s (v. 1.16.0) cumulative sum scaling (CSS) normalization [31]. Beta diversity (principle coordinate analysis, PCoA) was calculated on Bray-Curtis distances and tested for significance with ANOSIM (999 permutations) using vegan package in R (v. 2.4–1). Significant differential abundance (p <0.05) between samples was determined using DESeq2 (v. 1.14.1) at alpha = 0.05 [32] on OTUs >0.1% abundance. A core bacterial microbiome was determined comprising OTUs present in 100% of samples and a Venn diagram was generated with limma package [33] in R to visualize these data.

## Results

### Sequencing dataset

DNA extraction was completed on 132 samples. Despite many attempts, DNA obtained from SSO wrappers could not be PCR-amplified and these 18 samples were removed from analysis. As a result, a total of 6,206,143 sequencing reads were obtained from 114 samples (66 tobacco and 48 wrapper samples), for an average number of sequences per sample of 54,439 (+/- 23,473 SD). After sequencing, the minimum number of reads was 22 and the maximum was 104,223 across all samples. To ensure that samples included in the final dataset had appropriate sequence coverage, a Good’s coverage cutoff of 0.9 was chosen; samples falling below this cutoff (9 tobacco SSO samples and 1 wrapper SSC sample) were removed from the analysis (Figure A in S1 File). After Good’s filtering, the number of reads in the final dataset for wrapper samples ranged from 27,799 to 82,704 per sample, while the numbers of reads for tobacco samples ranged from 8,876 to 104,223 per sample.

Overall, sequences were clustered into 4,460 OTUs (97% identity) across all 104 samples: 2,689 OTUs from 57 tobacco samples, and 3,305 OTUs from 47 wrapper samples. After clean up (removing reads assigned to taxa ‘*Cyanobacteria*’ and OTUs with less than 10 reads), the total number of sequences used in downstream analyses was 5,540,948 from 104 samples, clustered into 2,681 OTUs.

### Microbiota differences between tobacco and wrapper samples

**Fig 1:**
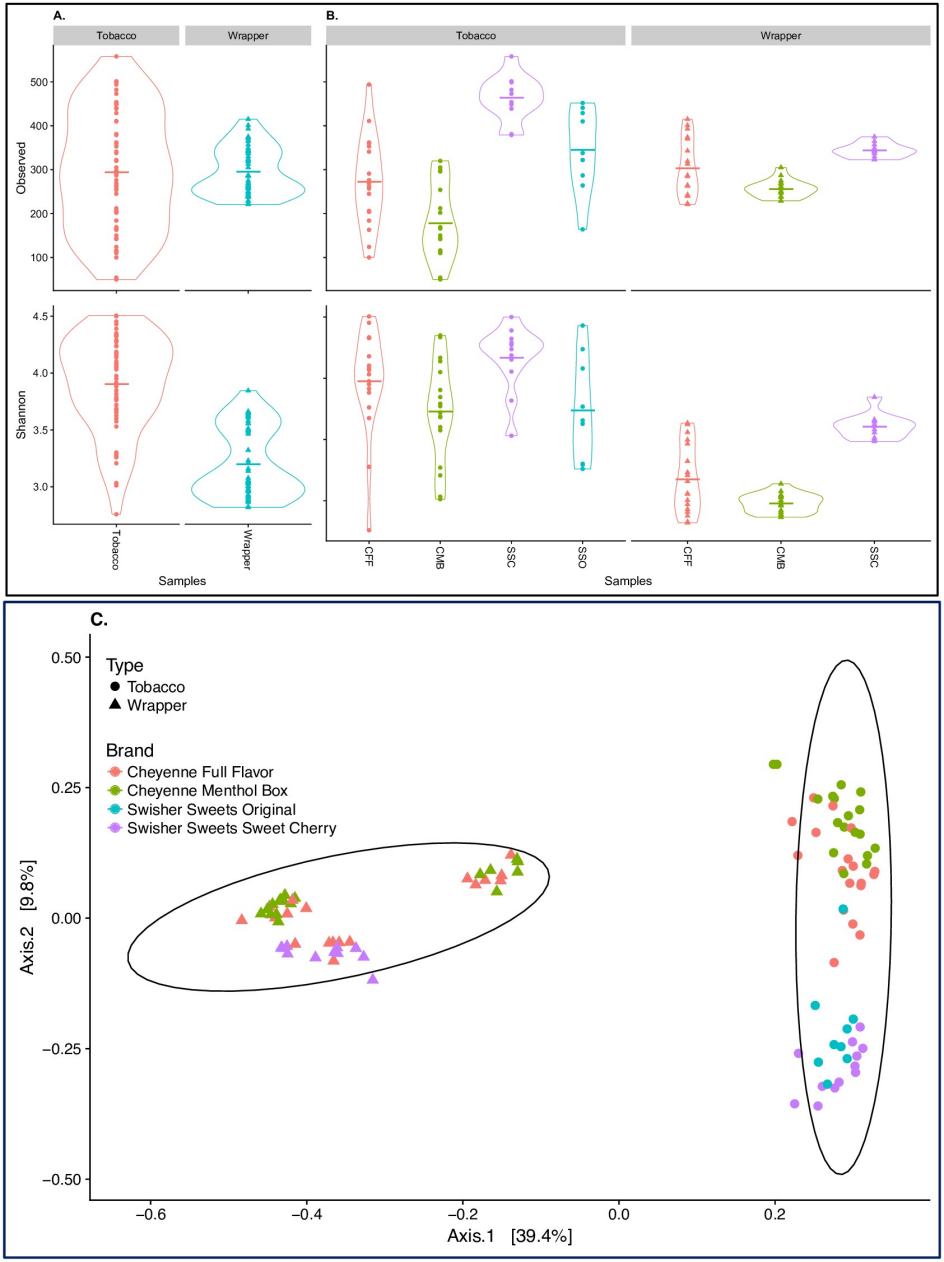
Bacterial diversity between tobacco and wrapper samples. Violin plots showing alpha diversity (Observed number of species and Shannon Index) [A] across all samples using rarefied data to minimum sampling depth (red, tobacco; and blue; wrappers); and [B] by product (red, Cheyenne Full Flavor (CFF); green, Cheyenne Menthol Box (CMB); purple, Swisher sweets cherry (SSC); and blue, Swisher sweets original (SSO). [C] PCoA analysis plots of Bray-Curtis computed distances between tobacco products. Sample type is denoted by shapes: triangle, wrapper; and circle, tobacco. Ellipses are drawn at 95% confidence intervals for product brand.

Differences in sequence coverage between samples can significantly affect measures of alpha diversity, and therefore, alpha diversity metrics (Shannon diversity and Observed species) were calculated on both rarefied (after down-sampling each sample to 7,964 reads) (Fig 1A) and non-rarefied data (Figure B in S1 File). Because analyses of rarefied and non-rarefied datasets yielded similar alpha-diversity results, only rarefied analyses are presented in Fig 1A and 1B. For both richness (Observed) and diversity (Shannon) metrics, bacterial communities from tobacco samples (Observed: 294.5 +/- 135.1; Shannon: 3.90 +/- 0.41) were significantly different (p<0.005) compared to those from wrapper samples (Observed: 295.0 +/- 54.2; Shannon: 3.20 +/- 0.30). Similarly, beta diversity analyses (Fig 1C) indicated that sample type (tobacco or wrapper) explained 96.7% (ANOSIM, p<0.005) of the variability observed in the bacterial communities, providing additional evidence that the bacterial microbiota of the tobacco samples is significantly different from that of the wrapper samples.

The top five bacterial phyla identified across all brands, lots and sample types (tobacco and wrapper) were *Firmicutes*, *Proteobacteria*, *Actinobacteria*, *Bacteroidetes* and *Chlorobi*. The tobacco samples were dominated by *Proteobacteria* compared to the wrapper samples. Specifically, the average relative abundance of *Proteobacteria* was 48.2% (SD: +/- 0.2; min: 27%; max: 71%) and 13.0% (SD: +/- 0.04; min: 10%; max: 17%) in tobacco and wrapper samples, respectively. In contrast, wrappers were dominated by *Firmicutes* at an average relative abundance of 83.9% (SD: +/- 0.6; min: 76%; max: 87%), compared to 39.8% (SD: +/- 0.15; min: 22%; max: 58%) in tobacco. The relative abundance of *Actinobacteria* was higher in tobacco, 10.6% (SD: +/- 0.06; min: 3%; max: 17%), when compared to that in wrappers, 0.6% (+/- 0). *Bacteroidetes* was found at a relative abundance of less than 3% in both tobacco and wrappers.

**Fig 2:**
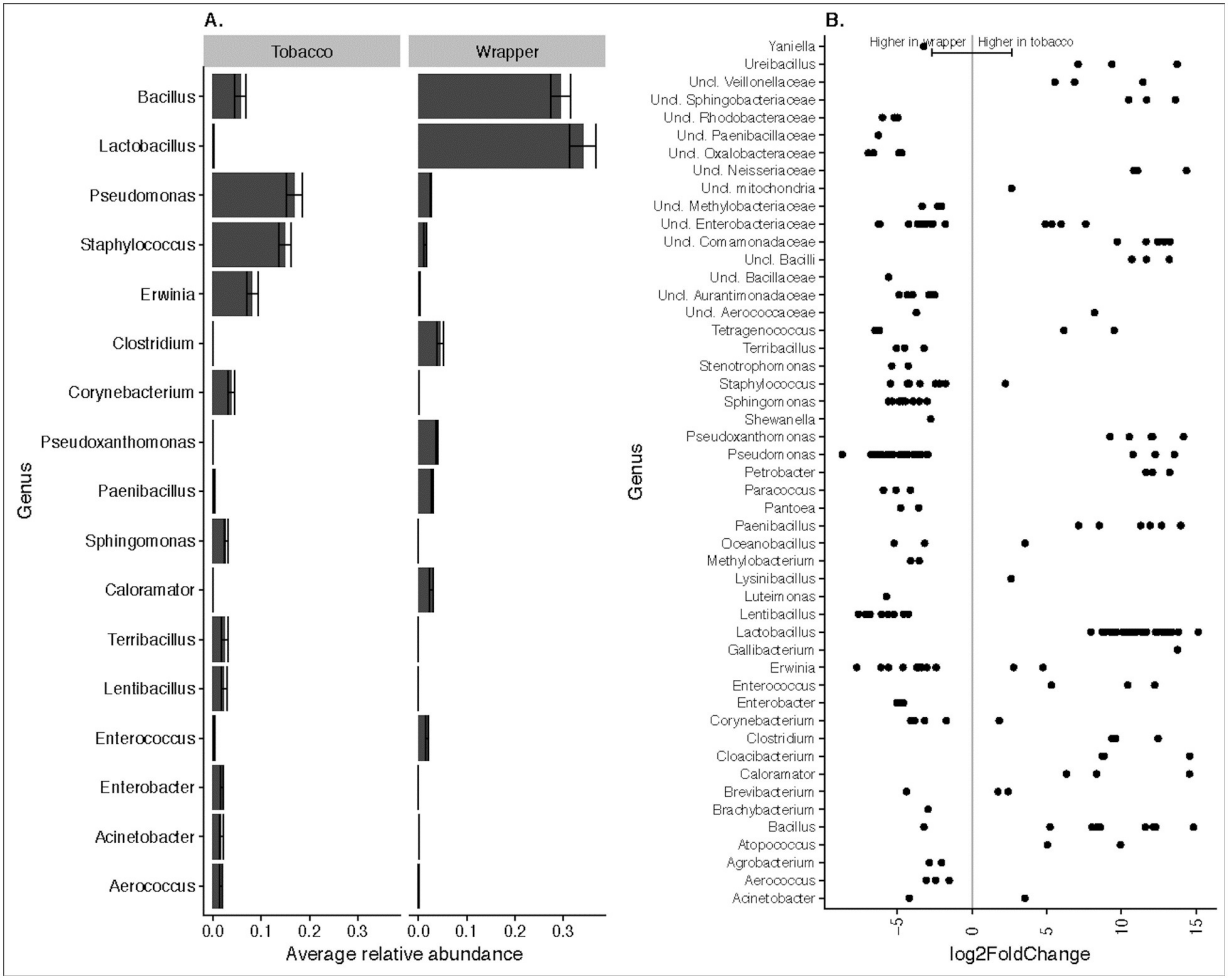
Overview of bacterial genera present in tobacco and wrappers. [A] Relative abundance of top 20 bacterial genera present in tobacco and wrapper samples. [B] Differential abundances of bacterial genera that were statistically significantly different (p < 0.05) between tobacco and wrapper samples. A positive log2-fold change value denotes an OTU that is significantly higher in tobacco, while a negative log2-fold change indicates an OTU that is significantly higher in wrapper. The grey line and arrows highlight the conversion in log2-fold change from negative to positive values.

In total, 2,035 OTUs were classified to the genus level, and among those, only 202 were identified to the species level. The top genera identified across all samples were *Bacillus*, *Lactobacillus*, *Staphylococcus*, *Pseudomonas*, *Pseudoxanthomonas*, *Clostridium*, *Corynebacterium*, *Lentibacillus*, *Pantoea* (Fig 2A). *Bacillus* and *Lactobacillus* dominated in the wrappers, while tobacco was dominated by *Pseudomonas* and *Staphylococcus* (Fig 2A). Differential abundances were also recorded for other bacterial genera, and those that were significantly different between tobacco and wrapper samples are shown in Fig 2B. At the species level, tobacco samples were characterized by a higher relative abundance of *Pantoea agglomerans*, *Shewanella algae*, *Bacillus clausii*, *Bacillus coagulans*, *Erwinia dispersa*, *Staphylococcus equorum*, *Tetragenococcus halophilus*, *Pseudomonas pseudoalcaligenes*, *Acinetobacter rhizosphaerae*, *Acinetobacter schindleri*, *Staphylococcus sciuri*, *Corynebacterium stationis*, and *Pseudomonas viridiflava*. Wrapper samples had a higher relative abundance of *Pseudomonas thermotolerans*, *Bacillus thermoamylovorans*, *Pseudoxanthomonas taiwanensis*, *Atopococcus tabaci*, *Petrobacter succinatimandens*, *Lactobacillus salivarius*, *Paenibacillus barengoltzii*, *Enterococcus cecorum*, and *Lactobacillus agilis*. The relative abundance of the top 100 species-level taxonomic assignments, across brands and sample types, is shown in S1 Table.

Despite these major differences in bacterial composition between little cigar tobacco and wrappers, a core microbiome analysis identified 68 OTUs that were present in all tobacco and wrapper samples. Within those, 8 OTUs (OTU# 2348- Firmicutes, OTU# 3747- *Bacillales*, OTU# 4279- *Paenibacillus sp*., OTU# 4318- Bacillales, OTU# 4667- *Pseudomonas thermotolerans*, OTU# 4897- Bacilli, OTU# 6365- *Pseudoxanthomonas taiwanensis*, OTU#6505- *Bacillus thermoamylovorans*) were identified as biomarkers for little cigar wrapper bacterial communities, i.e. only found in the wrapper samples and absent in the little cigar tobacco, while there weren’t any such OTUs exclusively present in the tobacco samples (Figure C in S1 File).

### Microbiota differences between brands

**Fig 3:**
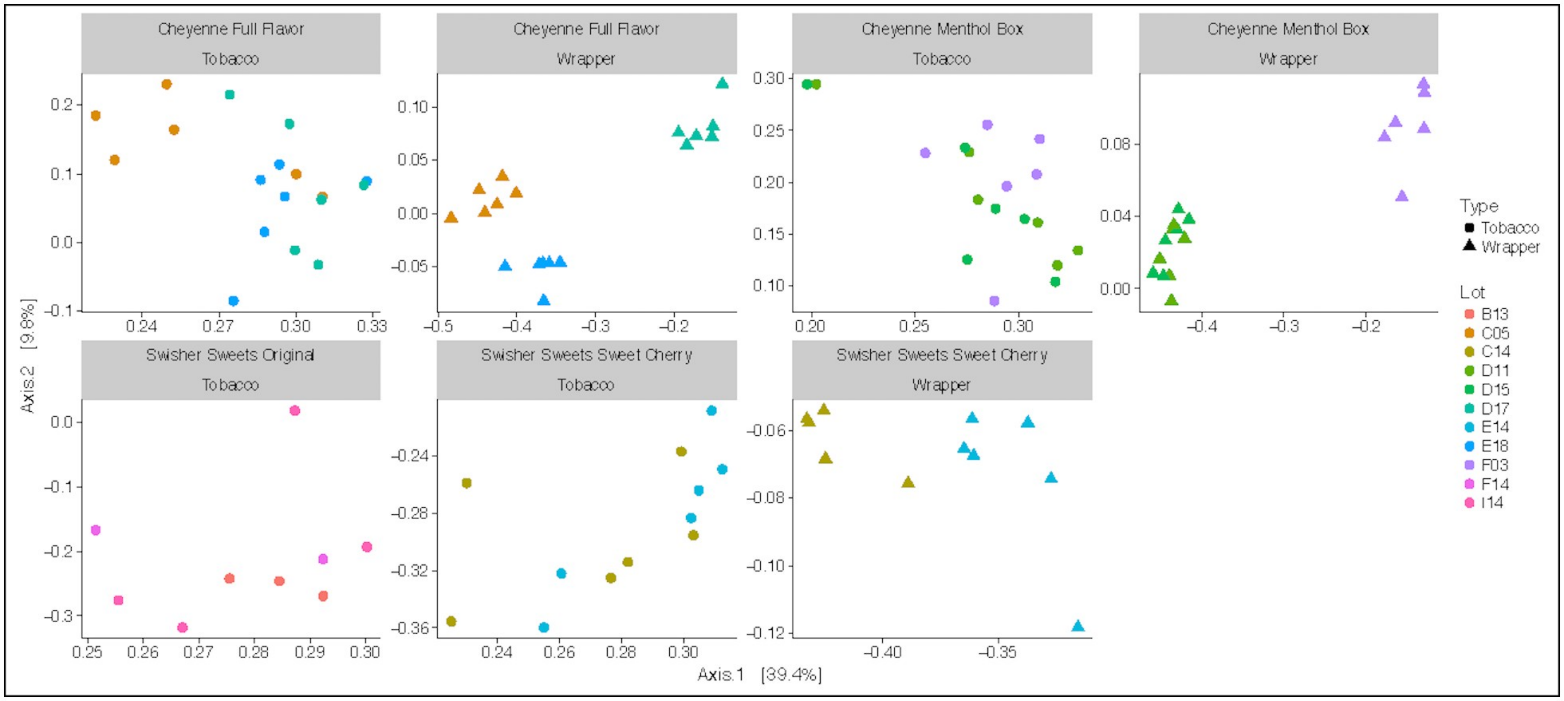
Microbial diversity between lots of different brands. PCoA analysis plots of Bray- Curtis computed distances between the lots of each brand and type. Type of product is denoted by shapes: triangle- wrapper and circle- tobacco. Colors denote the different lot numbers within each brand.

Overall, brand had a significant effect (p<0.005) on both the Shannon diversity metric and observed number of species. Observed number of species was higher in CFF and CMB wrappers compared to CFF and CMB tobacco, but lower in SSC wrappers compared to SSC tobacco (Fig 1B). Similarly, PCoA ordination showed significant differences in bacterial communities by brand (ANOSIM R = 0.138, p< 0.001) and lot (ANOSIM R = 0.134, p<0.001). Overall, the Cheyenne products cluster separately from the Swisher Sweets products (Fig 1C). Within each brand, the three lots showed significantly different bacterial communities in the wrapper samples (ANOSIM R = 0.134, p< 0.001), while they overlapped in the tobacco samples (Fig 3). UniFrac distances were used to measure the beta diversity between brands and sample type (Figure D in S1 File).

A comparison between brands showed that the relative abundance of *Actinobacteria* (SSO: 12.4% +/-0.08; SSC- 12% +/- 0.11) and *Firmicutes* (SSO: 60.7% +/-0.10; SSC: 58.3% +/- 0.19) was higher in Swisher Sweets tobacco compared to Cheyenne tobacco (*Actinobacteria*: CFF 8.1% +/-0.01; CMB- 3.3% +/- 0.0 and *Firmicutes*: CFF- 31.1.4% +/-0.06; CMB- 29.9% +/- 0.0). While a lower relative abundance of *Bacteroidetes* (SSO: 0.2% +/-0.0; SSC: 2.1% +/- 0.02) and *Proteobacteria* (SSO: 26.7.4% +/-0.06; SSC: 27.6% +/- 0.12) was found in Swisher Sweets tobacco compared to Cheyenne tobacco (*Bacteroidetes*–CFF: 1.7% +/-0.01; CMB: 1.9% +/- 0.01 and *Proteobacteria*- CFF: 59.1.4% +/-0.06; CMB: 71.9% +/- 0.01).

**Fig 4:**
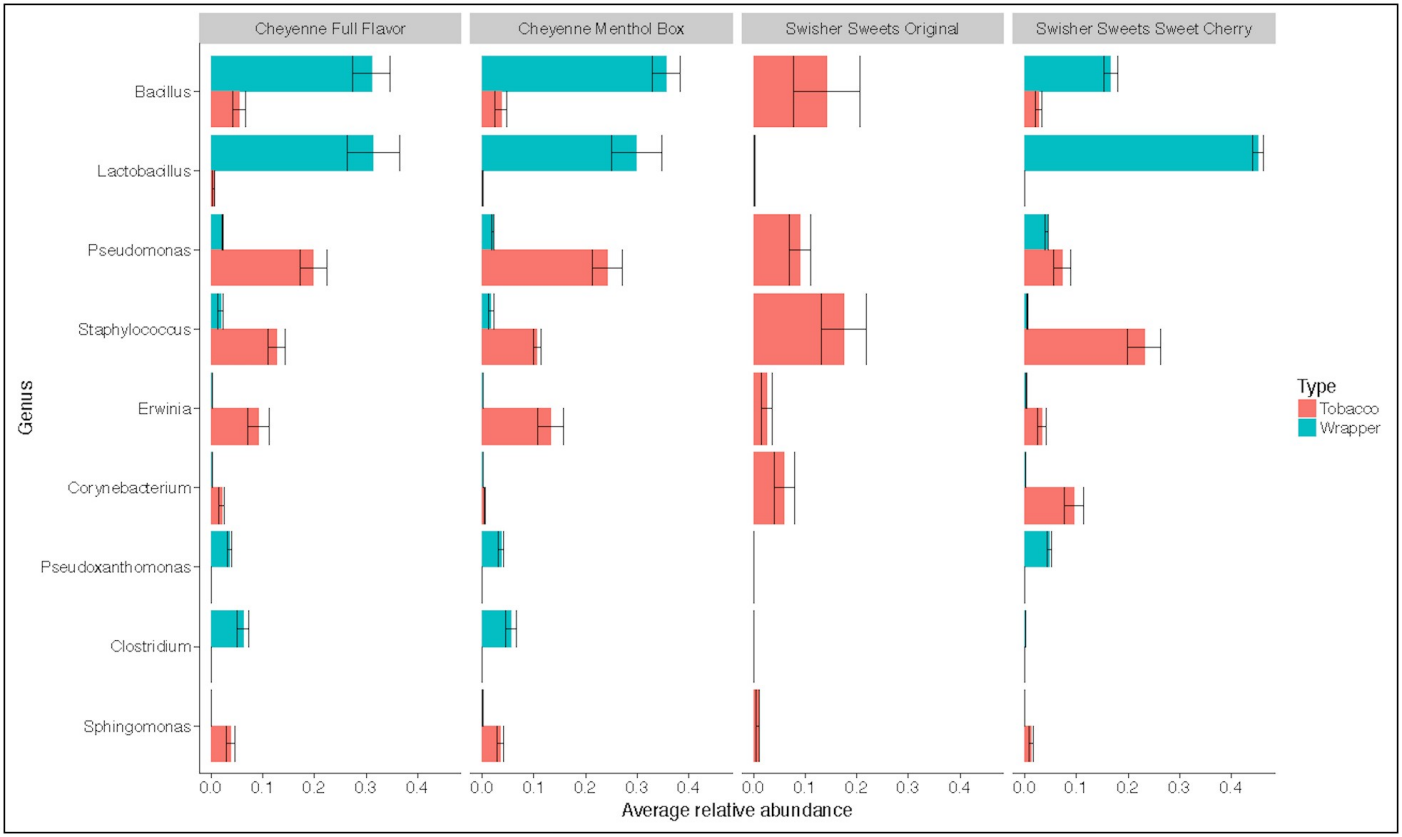
Box plots showing the top 10 bacterial genera that were found among all the four brands. Colors denotes the type of products: red- tobacco and blue- wrapper.

At the genus level, differences were also observed across brand (Fig 4). For instance, the average relative abundance of *Bacillus* in wrappers ranged from 16.8% in SSC to 35.8% in CMB, while in tobacco, average relative abundance of *Bacillus* was only 5.4% in CFF, 3.7% in CMB, 2.7% in SSC and 14.6% in SSO. Specifically, 30% of the *Bacillus* OTUs were assigned to *B*. *thermoamylovorans*, a spore-forming bacterium that possesses high-level heat resistance [34] and has been linked to food spoilage, particularly in the dairy industry.

**Fig 5:**
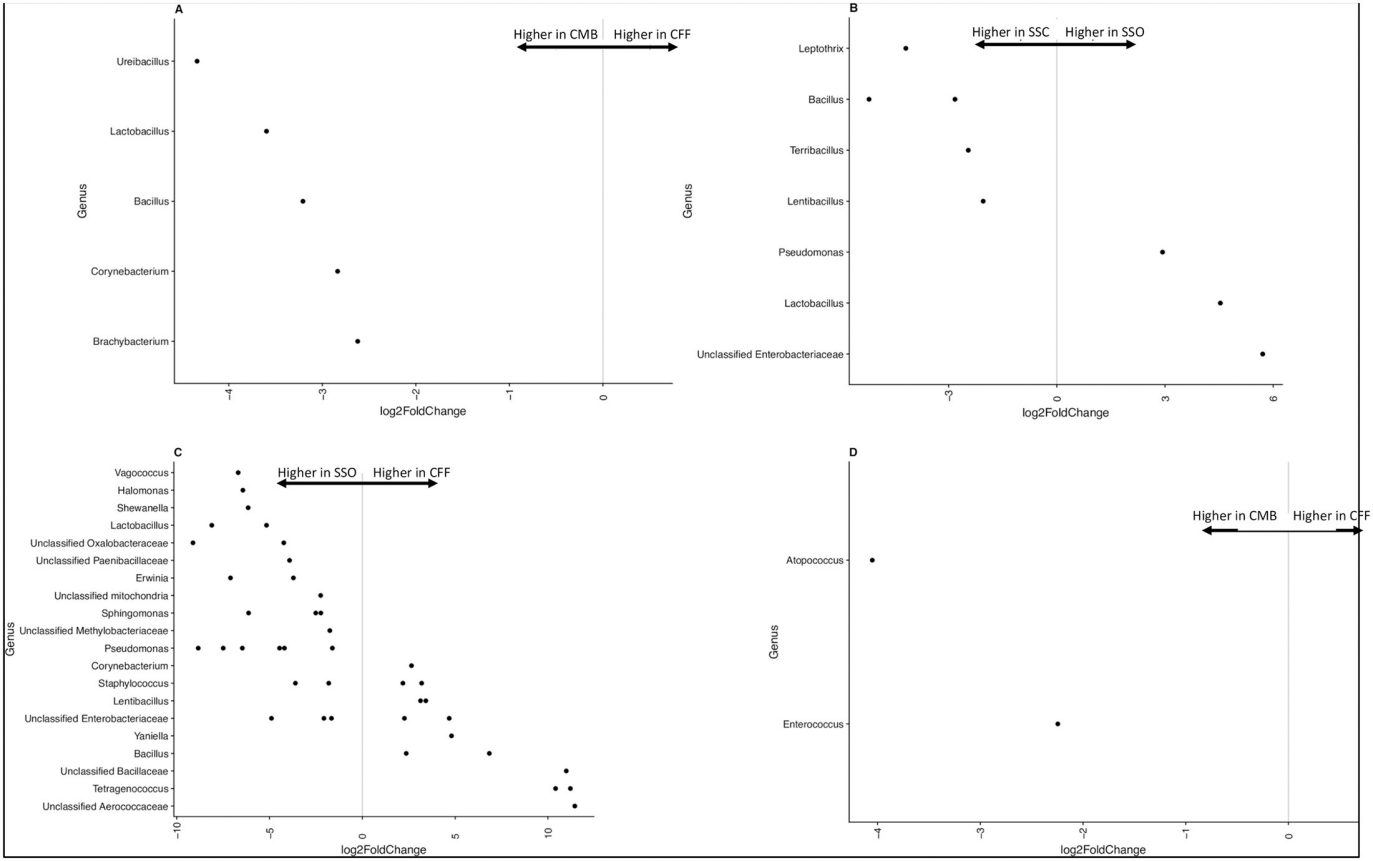
Differential abundance plots of bacterial genera that were statistically significantly different (p< 0.05) between the brands. The bacterial OTUs that could not be assigned to a genus are represented as “Unclassified” and are assigned to their family level. The grey line and arrows highlight the conversion in log2-fold change from negative to positive values. [A] Comparing Cheyenne Menthol Box (CMB) to Cheyenne Full Flavor (CFF) tobacco samples. A positive log2-fold change value denotes an OTU that is significantly higher in CFF, while a negative log2-fold change indicates an OTU that is significantly higher in CMB. [B] Comparing Swisher sweets cherry (SSC) to Swisher sweets original (SSO) tobacco samples. A positive log2-fold change value denotes an OTU that is significantly higher in SSO, while a negative log2-fold change indicates an OTU that is significantly higher in SSC. [C] Comparing Swisher sweets original (SSO) to Cheyenne Full Flavor (CFF) tobacco samples. A positive log2-fold change value denotes an OTU that is significantly higher in CFF, while a negative log2-fold change indicates an OTU that is significantly higher in SSO. [D] Comparing Cheyenne Menthol Box (CMB) to Cheyenne Full Flavor (CFF) wrapper samples. A positive log2-fold change value denotes an OTU that is significantly higher in CFF, while a negative log2-fold change indicates an OTU that is significantly higher in CMB.

Comparing only tobacco samples, a statistically significantly lower relative abundance of the bacterial genera *Brachybacterium*, *Corynebacterium*, *Bacillus*, *Lactobacillus* and *Ureibacillus* was found in CMB when compared to CFF (Fig 5A). Comparison of tobacco bacterial communities from SSO and SSC showed a statistically significantly (p<0.001) higher relative abundance of *Lactobacillus* and *Pseudomonas* in SSO, and a lower relative abundance of *Lentibacillus*, *Terribacillus*, *Bacillus* and *Leptothrix* in SSC (Fig 5B). Comparing between tobacco samples from SSO and CFF, *Corynebacterium*, *Lentibacillus*, *Yaniella*, *Bacillus* and *Tetragenococcus* were found to be at a significantly higher relative abundance in CFF samples (Fig 5C). While comparing wrapper samples, a lower relative abundance of *Enterococcus* and *Atopococcus* was seen in CMB compared to CFF (Fig 5D).

## Discussion

This study provides novel evidence that the bacterial communities of little cigars and cigarillos are not only diverse, but also differ significantly between the tobacco and the wrapper, and across brands. During the manufacturing process of a general cigar, different types of leaves are used for making the wrapper, filler and binder. These leaves may be grown in multiple regions of the world, under differing soil and climatic conditions [35]. This manufacturing process, along with differences in the composition of the materials within the tobacco versus the wrapper, likely contributed to the observed variability between these sample types. Tobacco samples also differed in bacterial community composition across brands, which is not unexpected considering that brands are manufactured under different industrial conditions with variable tobacco blends [36]. This can also have an effect on the individual lots that are manufactured, resulting in the variable bacterial community composition that we observed in wrapper samples across lots in this study.

Interestingly, a significantly higher abundance of the bacterial genera *Lentibacillus* spp., *Terribacillus* spp., *Bacillus* spp. and *Leptothrix* spp. was found in the Swisher Sweets Cherry tobacco samples when compared to Swisher Sweets Original tobacco samples. The predominance of these four genera in the flavored little cigars may be due to the effect of flavoring of the product itself. Even though hundreds of flavors of little cigars are available on the market, not much is known about the chemical constituents of the flavoring additives and their associated impacts on tobacco microbiology and human health. In contrast, a few studies have evaluated the chemicals used to flavor electronic cigarettes including diacetyl and acetyl propionyl, two chemicals that are known to cause bronchiolitis obliterans [37–39]. In September 2009, FDA banned the use of flavoring (other than menthol) in cigarettes in an effort to discourage smoking in the US (particularly among youth and young adults), but this was not imposed on other tobacco products like little cigars and electronic cigarettes. In the present study, mentholated little cigars (CMB) were characterized by a significantly higher abundance of *Brachybacterium* spp., *Corynebacterium* spp., *Bacillus* spp., *Lactobacillus* spp., *Ureibacillus* spp., *Enterococcus* spp. and *Atopococcus* spp. when compared to non-mentholated little cigars (CFF). Menthol has antimicrobial properties and the presence of this flavor may have directly shifted the bacterial communities within the mentholated products [16]. Mentholation of cigarettes also has been shown to pose a greater threat in causing cancers and other respiratory diseases when compared to non-mentholated cigarettes [40,41]. The relationship between mentholation, modification of little cigar bacterial community composition and their potential synergism in the development of adverse health effects among users of these products is an area of future exploration.

Future work focusing on the potential human health effects associated with bacterial pathogens identified in the tested little cigar products is also warranted. Here, we identified several potential pathogens in tested products. For instance, *Pseudomonas pseudoalcaligenes* and *Staphylococcus sciuri* were detected at a high relative abundance in little cigar tobacco samples. Previous studies have shown that *P*. *pseudoalcaligenes* is a potential causative agent of human infections [42], including peritonitis (inflammation of the inner wall of the abdomen) [43] and *S*. *sciuri* is an emerging human pathogen causing endocarditis [44], urinary tract infections [45], pelvic inflammatory diseases [46], wound infections [47] and septic shock [48]. *Pantoea agglomerans*, *Shewanella algae* and *Acinetobacter schindleri* were also found at a higher relative abundance in tobacco samples compared to wrapper samples. *P*. *agglomerans* is a known pathogen causing bone joint, blood and soft tissue infections [49], and a Danish study isolated *S*. *algae* from 67 ear infection cases [50]. *Acinetobacter spp*. are commonly isloated from hospital environments [51] and *A*. *schindleri* is regarded as an opportunistic pathogen [52].

Among the other bacterial species identified in little cigar products that may be relevant to users’ health, *Lactobacillus salivarius* and *L*. *agilis* were found at a significantly higher abundance in the tobacco samples from Swisher Sweets Original and Cheyenne Menthol Box. Even though infections with *L*. *salivarius* are uncommon, this organism was found to be the causative agent for diabetic ketoacidosis accompanied by bacteremia, emphysema and respiratory failure in one reported case [53]. In addition, wrapper samples from Cheyenne Full Flavor had a significantly higher relative abundance of *Enterococcus cecorum*. *E*. *cecorum* has been previously shown to be associated with bacteremic peritonitis in humans [54].

Nevertheless, in order for potential pathogens present in little cigars to impact the health of users, these microorganisms would first have to survive the combustion process and then be transferred to the upper respiratory system via mainstream smoke. While the tip of combustible tobacco products (the combustion zone) can reach up to 900°C, immediately downstream of the combustion zone is the “cooler pyrolysis/distillation zone” where the majority of chemical constituents in smoke are generated [55] and “the temperature of the aerosol drops rapidly to slightly above room temperature as it travels down the tobacco rod,” [56]. This area correlates with the portion of the product that is held between fingers to the portion that is inserted into the mouth, all of which remain at temperatures that are comfortable to the touch, comfortable to inhale and could accommodate live bacteria. Larsson et al. (2008) showed that the mainstream smoke of cigarette tobacco that was spiked with *Escherichia coli* cells had a 4-fold higher concentration of lipopolysaccharides (a key component of the outer membranes of gram-negative bacteria) compared to that of cigarette tobacco that was not spiked [57,58]. Pauly et al. (2010) demonstrated that differing microbial constituents, including lipopolysaccharides, of tobacco eluates activated human lung macrophage, producing pro-inflammatory factors such as IL-8. These data suggest that bacterial constituents may be transferred to users via mainstream smoke, and that “lung inflammation of long-term smokers may be attributed in part to tobacco-associated bacterial and fungal components that have been identified in tobacco and tobacco smoke” [58]. Moreover, ongoing culture-based experiments in our lab are providing the first evidence that viable bacteria can survive the combustion process and be recovered from cigarette mainstream smoke (data not shown). Additional studies are necessary to further explore whether viable microorganisms inhaled within mainstream smoke are able to colonize the upper respiratory tracts of users, contribute to lung inflammation, and potentially influence health outcomes.

In summary, our findings suggest that the bacterial microbiota of little cigar tobacco and wrappers is diverse and distinct, and bacterial communities vary across brands. Strengths of this study include the sample size, the ability to demonstrate replication of findings across biological replicates and thorough statistical analysis of the sequencing data. As with all 16S rRNA-based sequencing studies, this study had limitations, including inherent PCR amplification biases, our limited ability to assign species-level classifications, our inability to discriminate between the live/active and dead proportions of the detected bacterial communities, and the lack of data regarding viability of detected bacteria. However, previous work demonstrates that bacteria can be cultured from smokeless tobacco products [18], therefore, it is likely that the same would be true for little cigars and cigarillos. One additional limitation is that this study included samples from one time point only, providing only a snapshot of the bacterial communities present in the tested products. Longitudinal studies of these products placed under different storage conditions are ongoing in our group and will provide insights into whether these bacterial communities are dynamic, changing in the period of time between the sale and use of these products. Finally, future studies that include functional analyses, in addition to taxonomic data, would further our overall understanding of the microbiota of little cigars and cigarillos.

## Supporting information

S1 File **Figure A. Good’s Coverage across all tobacco and wrapper samples. Figure B. Alpha diversity within tobacco and wrapper samples (generated with non-rarefied data) measured using Observed OTUs and the Shannon Index**. Color denotes product brand: Cheyenne full flavor 100’s (CFF), red; Cheyenne menthol flavor 100’s (CMB), green; Swisher sweets little cigars sweet cherry flavor (SSC), purple; and Swisher sweets cigarillo natural sweet (SSO), blue. **Figure C. Core microbiome analysis indicating the number of observed taxonomic units (OTUs) shared between tobacco and wrapper samples**. **Figure D. Principle coordinate analysis (PCoA) using unweighted Unifrac distances.** Tobacco samples are represented by circles, and wrapper samples are represented by triangles. Differing colors denote the various lots tested.
(DOCX)

S1 Table Relative abundance of top 100 bacterial species identified across all brands and type of tobacco products. (XLSX)
