# Supplementary material for: Little cigars and cigarillos harbor diverse bacterial communities that differ between the tobacco and the wrapper
Source: PLoS One. 2019 Feb 22;14(2):e0211705. doi: 10.1371/journal.pone.0211705 (PMC6386278; doi:10.1371/journal.pone.0211705)
Supplement: S1 File — Figure A. Good’s Coverage across all tobacco and wrapper samples. Figure B. Alpha diversity within tobacco and wrapper samples (generated with non-rarefied data) measured using Observed OTUs and the Shannon Index. Color denotes product brand: Cheyenne full flavor 100’s (CFF), red; Cheyenne menthol flavor 100’s (CMB), green; Swisher sweets little cigars sweet cherry flavor (SSC), purple; and Swisher sweets cigarillo natural sweet (SSO), blue. Figure C. Core microbiome analysis indicating the number of observed taxonomic units (OTUs) shared between tobacco and wrapper samples. Figure D. Principle coordinate analysis (PCoA) using unweighted Unifrac distances. Tobacco samples are represented by circles, and wrapper samples are represented by triangles. Differing colors denote the various lots tested. (DOCX) [file pone.0211705.s001.docx]

**Figure A:** Good’s Coverage across all tobacco and wrapper samples.

**Figure B:** Alpha diversity within tobacco and wrapper samples (generated with non-rarefied data) measured using Observed OTUs and the Shannon Index. Color denotes product brand: Cheyenne full flavor 100’s (CFF), red; Cheyenne menthol flavor 100’s (CMB), green; Swisher sweets little cigars sweet cherry flavor (SSC), purple; and Swisher sweets cigarillo natural sweet (SSO), blue.

**Figure C:** Core microbiome analysis indicating the number of observed taxonomic units (OTUs) shared between tobacco and wrapper samples.

**Figure D:** Principle coordinate analysis (PCoA) using unweighted Unifrac distances. Tobacco samples are represented by circles, and wrapper samples are represented by triangles. Differing colors denote the various lots tested.
